# Epidemiological and spatial analysis of newly diagnosed HIV-1/AIDS patients before antiretroviral therapy in Ningxia from 2020 to 2021

**DOI:** 10.1371/journal.pone.0322389

**Published:** 2025-04-22

**Authors:** Yichang Liu, Xiaofa Ma, Jianxin Pei, Dongzhi Yang, Yufeng Li, Xiaohong Zhu, Zhonglan Wu

**Affiliations:** 1 School of Public Health, Ningxia Medical University, Yinchuan, China; 2 Ningxia Hui Autonomous Region Centers for Disease Control and Prevention, Yinchuan, China; Institut Pasteur Cambodia: Institut Pasteur du Cambodge, CAMBODIA

## Abstract

The high mutability of human immunodeficiency virus type 1 (HIV-1) and the widespread use of antiretroviral drugs have rendered genetic diversity and pre-treatment drug resistance (PDR) significant obstacles to the success of antiretroviral therapy (ART). However, the research on the epidemiological and spatial distribution characteristics of PDR in Ningxia is still insufficient. A cross-sectional study utilized pre-treatment blood samples collected between 2020 and 2021 from the biorepository in May 2024. Partial pol gene sequences were obtained through plasma collection and RNA extraction. Drug resistance analysis was performed using the Stanford University HIVdb algorithm. Molecular network were constructed using Cytoscape 3.10.0. Spatial analysis and visualization were further conducted using ArcGIS10.8.1. 95 sequences were obtained, among which 7 HIV-1 genotypes were detected and CRF07_BC (67.37%, 64/95) was the predominant one. Drug resistance mutations (DRMs) were detected in 13.68%(13/95) of the sequences. The risk of PDR occurrence was higher among individuals with CRF07_BC strain types. The 24 sequences of CRF07_BC, CRF01_AE, and URF subtypes grouped into nine transmission clusters in the molecular network, with CRF07_BC showing the highest integration and clustering rates. HIV-1 infections resistant to PDR were observed in all five cities in NHAR, accompanied by cross-city transmission. Additionally, seven imported sequences were detected, comprising CRF07_BC, CRF01_AE, and C subtypes, along with three sequences of CRF55_01B with high similarity to nonlocal sequences. From 2020 to 2021, the HIV-1 diversity increased significantly in NHAR, with the prevalence of PDR reaching moderate levels and evidence of resistance transmission. The districts and counties under the jurisdiction of Yinchuan City emerged as hotspots for both pre-treatment HIV/AIDS patients and the distribution of resistant strains. It is imperative to enhance PDR testing and implement targeted interventions in key areas to minimize the emergence and dissemination of resistant virus variants.

## Introduction

Since the first domestic was diagnosed with HIV-1/acquired immune deficiency syndrome (AIDS) [1], HIV-1 has evolved rapidly in China over 30 years, with an increasing number of infected individuals and increased genotype complexity [2]. At the end of 2022, there were 1.223 million people living with HIV-1 (PLWH) currently alive, with 689000 being HIV-infected and 534000 being AIDS patients [3]. The prevalence of AIDS has become a tremendous challenge to public health in China. Since the Chinese government implemented the National Free Antiretroviral Treatment Program (NFATP) in 2003 [4], ART has exhibited remarkable effectiveness in suppressing HIV-1 viral replication, leading to a notable reduction in plasma viral load (VL) and subsequently enhancing the functionality of the immune system [5,6]. However, with the proportion of the population who receive ART gradually expanded, the occurrence of drug resistance became the main reason of the failure of PLWH antiretroviral therapy, which results in the spread of resistant strains [7]. The new problem poses a great challenge to the controlling of the HIV epidemic. Thus, it is important to know the prevalence of drug resistance in the population before ART, to provide baseline information for effective control of HIV prevalence. PDR denotes the established presence of drug resistance prior to commencing ART, encompassing transmitted drug resistance(TDR), primary resistance, and the reinitiation of antiviral therapy following a previous treatment regimen [8]. World Health Organization(WHO) formulated a policy of targeting HIV-1 drug resistance in 2012 [9], and made a monitoring method for starting ART drug resistance population, recommending monitoring the prevalence of HIV-1 drug resistance and risk factors associated with HIV-1 drug resistance. On account of the HIV-1 has the character of easy mutation, using the routing drug resistance monitoring of HIV-1 gene sequences to construct the transmission network, which could effectively monitor the transmission of HIV-1 and analyze the transmission characteristics of the virus among PLWH [10–12]. Spatial autocorrelation analysis can effectively delineate the distribution patterns and identify hotspots of HIV-1/AIDS cases within a local region, thereby guiding targeted policy interventions and enhancing the efficiency of prevention and control efforts.

NHAR is located in the inland region of Northwest China, with low prevalence of HIV-1. However, amidst the proliferation of the internet, the way that PLWH establish social connections has become increasingly concealed or private. Molecular transmission network provide insights into the discovery of the HIV-1 transmission chains and help pinpoint potential transmission pathways and vulnerable populations. The combined application of epidemiological investigation and spatial analysis has established a multidimensional framework for tracing and interrupting HIV-1 transmission chains, as well as analyzing its geographical distribution. This framework provides valuable insights into depicting viral transmission pathways and identifying potential high-risk regions and populations. It supports enhanced primary prevention efforts within these areas, aiming to interrupt transmission routes and protect vulnerable populations.

Unfortunately, the research in this area exploring the PDR of HIV-1 in NHAR remains scarce. This study selected the HIV-1 infected individuals newly diagnosed in NHAR between 2020 and 2021 who were ART-naive. The primary purpose of this study is to investigate and analyze the prevalence and geographical distribution of PDR in NHAR. This endeavor strives to elucidate the epidemiological and spatial patterns associated with PDR mutations in NHAR, offering crucial insights into the dynamic landscape of HIV-1 resistance and its implications for local health policies and clinical practices.

## Materials and methods

### Study participants

A cross-sectional study was initiated in May 2024, utilizing blood samples from a pre-treatment population newly diagnosed during the period from 2020 to 2021, sourced from the biorepository of the Ningxia Center for Disease Control and Prevention. 95 patients with HIV-1 infection were enrolled according to the following criteria:(1) newly diagnosed HIV-1 cases; (2) had not received ART before enrollment; (3) blood drawing successfully; (4) agreed to participate in the survey and signed an informed consent(the data were analyzed anonymously); (5) viral load of ≥400 copies/mL. Prior to the enrollment, participants’ demographic information was obtained from the Ningxia Hui Autonomous Region AIDS comprehensive response information management system.

### Laboratory tests

Blood plasma samples were preserved at -80°C before RNA extraction. Viral RNA was extracted from plasma utilizing the automated nucleic acid extraction and purification system, along with the HIV-1 viral load assay kit, both sourced from Zhuhai Livzon Diagnostics Inc. according to the manufacturer protocol. Partial pol sequence that corresponded to codons 1–99 of protease and codons 1–299 of reverse transcriptase was amplified by in-house nested reverse transcription PCR. The cDNA synthesis and nested PCR operation were performed with a One Step RNA PCR Kit(Takara, China). The primers used for RT-PCR and Nested PCR are detailed in Table 1. The cycling conditions involved the following steps: an initial incubation at 55°C for 45 minutes, followed by 94°C for 2 minutes. Then, 35 cycles were performed, each consisting of 94°C for 15 seconds, 50°C for 20 seconds, and 72°C for 2 minutes. After the cycles, a final extension was conducted at 72°C for 10 minutes, and the products were kept at 4°C for storage. The amplified products were analyzed using 1% agarose gel electrophoresis to observe the bands, and positive products were selected for gene sequencing at SinoGenoMax Co., Ltd. (Beijing China).

**Table 1:** Primers used for RT-PCR and nested PCR.

Item	Primer Name	Sequence (5’ to 3’)
RT-PCR	MAW26	TGGAAATGTGGAAAAGAAGGAC
	RT21	CTGTATTTCAGCTATCAAGTCTTTTGATGGG
Nested PCR	PRO1	CAGAGCCAACAGCCCCACCA
	RT20	CTGCCAATTCTAATTCTGCTTC

### Identification of HIV-1 genotypes

Utilizing the BLAST tool from the HIV-1 database (https://www.hiv.lanl.gov/), the genetic subtype was initially identified. For those gene sequences that remained inconclusive, the online tools RIP and jpHMM from the HIV-1 Database were employed for preliminary recombination analysis. Subsequently, RDP4 and Simplot software were utilized to precisely locate the recombination breakpoints. Afterward, the sequences were aligned and adjusted using Clustal W in MEGA 11 software. Finally, a phylogenetic tree was constructed employing the Neighbor-Joining method with a Bootstrap value of 1000, allowing for the determination of the HIV-1 genetic subtype and a comprehensive analysis of its epidemiological characteristics.

### Genotypic resistance analysis

DRMs and resistance levels were determined based on the Stanford HIVdb Program (https://hivdb.stanford. edu/hivdb/by-sequences/). The database classifies the degree of DRMs into five levels: susceptible (S), potential resistance (P), low-level resistance (L), intermediate resistance (I), and high-level resistance (H). In this study, the presence of low-level or higher resistance to any drug is considered as genotypic resistance.

### Molecular transmission network

Cytoscape 3.10.0 was used to establish the HIV-1 molecular transmission network, which is based on a pairwise genetic distance threshold under the Tamura-Nei (TN93) nucleotide substitution model. The pilot analysis was conducted with a range of pairwise genetic distances spanning from 0.1% to 1.5%, incremented in 0.2% intervals, for comprehensive evaluation.

### Spatial analysis

The spatial distribution and clustering of HIV/AIDS patients in NHAR’s counties are explored by the application of general spatial autocorrelation. The Moran’s I index (-1–1) assesses HIV-1/AIDS distribution in NHAR. A positive Moran’s I > 0 with Z-score > 1.96 indicates clustering (positive autocorrelation). Conversely, a negative Moran’s I < 0 with Z-score < -1.96 signifies dispersion (negative autocorrelation). When neither applies, infections are randomly distributed [13]. Subsequently, this study employed local autocorrelation analysis to identify local clusters that could not be discerned through spatial autocorrelation alone. The Getis-Ord Gi* statistic was utilized to calculate z-scores and P-values; local clusters with z-scores > 1.96 were defined as hotspots, indicating higher concentrations of infections in those areas, while local clusters with z-scores < -1.96 were designated as coldspots. ArcGIS10.8.1 software was used for spatial analysis and map creation.

### Statistics analysis

Statistical analysis was performed using Stata MP17 (StataCorp, USA)and Excel 2010. All selected demographic and laboratory data were subjected to descriptive statistical analysis. Categorical variables were analysis represented as percentages. Chi-square test or Fisher exact test was used to compare proportions between different groups. Univariate and multivariate logistic regression models were used to analyze the potential risk factors. All tests were two-tailed and *P* values < 0.05 were considered as statistically significant.

### Ethics and consent

The research protocols of the present study were approved by the Institutional Review Board of the Ningxia Hui Autonomous Region Center for Disease Control and Prevention(No.2024-LLSC-088).

## Results

### Participant characteristics and genotypes distribution

**Fig 1:**
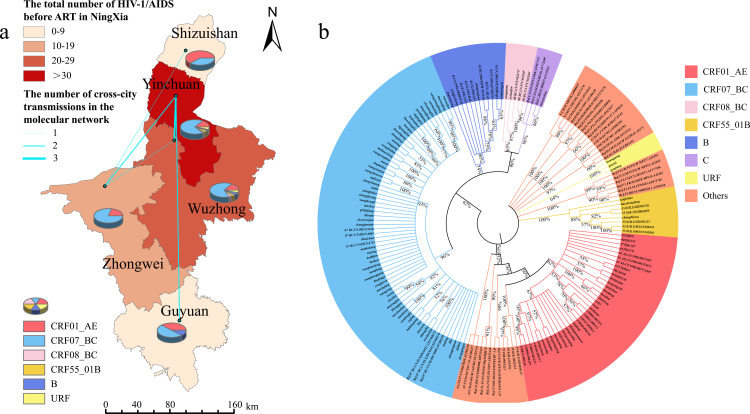
a. The distribution and quantitation of genotypes of HIV-1 in NHAR. (Note: The base map is from the China National Bureau of Surveying, Mapping and Geoinformation which provides a standard map download service, Review No. GS(2024)0650). b. The phylogenetic tree of newly diagnosed HIV-1 infected people before ART in NHAR.

95 sequences were successfully acquired and included in the analyses, and the sequences have been uploaded to the NCBI database (Genbank: PV170438-PV170532). Among the sampled individuals, 88 were geographically distributed across five cities within NHAR, while the remaining seven individuals hailed from various provinces outside this region. Notably, the majority of HIV-1 infected persons resided in the urban areas of Yinchuan and Wuzhong city. (Fig 1a). Among them, the majority ranged from 31 to 50 years (43.16%). There were 85.26% and 14.74% males and females, respectively. The transmission routes were complicated and included heterosexual transmission (58, 61.05%), homosexual transmission (36, 37.90%), and injecting drug use (1, 1.05%). Statistically significant associations were observed between gender and subtypes (*P*=0.034) and between age group and subtypes (*P*=0.032). (Table 2).

**Table 2:** Demographic characteristics and genotypes of 95 HIV-1 infected persons without ART.

Characteristic	Total (%)	HIV-1 Genotype							*P* value
		CRF01_AE	CRF07_BC	CRF08_BC	CRF55_01B	B	C	URFs	
**Gender**									0.034^a^
Male	81(85.26%)	16	57	1	3	2	0	2	
Female	14(14.74%)	2	7	1	0	2	1	1	
**Age**									0.032^a^
~30	20(21.05%)	4	14	0	1	1	0	0	
31~	41(43.16%)	3	31	0	1	3	0	3	
51	34(35.79%)	11	19	2	1	0	1	0	
**Residence**									0.540^a^
Yinchuan	46 (48.42%)	6	34	1	2	1	0	2	
Shizuishan	5 (5.26%)	3	2	0	0	0	0	0	
Wuzhong	22 (23.16%)	3	15	1	1	1	0	1	
Guyuan	5 (5.26%)	1	4	0	0	0	0	0	
Zhongwei	10 (10.53%)	4	5	0	0	1	0	0	
Other cities and provinces	7 (7.37%)	1	4	0	0	1	1	0	
**Marital status**									0.994^a^
Unmarried	23 (24.21%)	5	16	0	1	1	0	0	
Married	43 (45.26%)	7	30	1	1	1	1	2	
Divorced or widowed	29 (30.53%)	6	18	1	1	2	0	1	
**Degree of education**									0.735^a^
Illiterate	8 (8.42%)	2	6	0	0	0	0	0	
Primary school	14 (14.74%)	5	8	0	0	0	1	0	
Junior high school	31 (32.63%)	4	21	2	2	2	0	0	
High school or technical school	22 (23.16%)	5	14	0	1	1	0	1	
College or above	20 (21.05%)	2	15	0	0	1	0	2	
**Route of infection**									0.797^a^
Homosexual transmission	36 (37.90%)	6	27	1	1	0	0	1	
Heterosexual transmission	58 (61.05%)	12	36	1	2	4	1	2	
Injecting drug use	1 (1.05%)	0	1	0	0	0	0	0	
**Drug resistance**									0.062^a^
Yes	13 (13.68%)	1	8	0	2	2	0	0	
No	82 (86.32%)	17	56	2	1	2	1	3	

^a^*P* values were obtained from Fisher test.

7 genotypes were identified, and the phylogenetic tree of the pol region was shown (Fig 1b). Among these genotypes, the most common genotype was CRF07_BC (67.37%, 64/95), followed by CRF01_AE (18.94%, 18/95). The other genotypes were subtype B (4.21%, 4/95), CRF55_01B (3.16%, 3/95), CRF08_BC (2.11%, 2/95), subtype C (1.05%, 1/95). The genotypes were plentiful in Yinchuan and Wuzhong cities (Fig 1a). CRF07_BC was distributed mostly in the central and western NHAR and was the predominant strain in Yinchuan, Wuzhong, Zhongwei, and Guyuan City. However, the proportion of CRF01_AE was higher than CRF07_BC in Northern NHAR, which was the predominant strain. CRF08_BC, CRF55_01B, URF, and subtype B were identified in the central and western NHAR, among them CRF08_BC, CRF55_01B, URF was only detected in Yinchuan and Wuzhong city. We selected the most critical parameters for direct comparisons, including subtype and age (Table 3), subtype and transmission routes (Table 4), subtype and drug resistance (Table 5), two major prevalent subtypes and different routes of infection, two major prevalent subtypes and drug resistance, and drug resistance and age, as described in the S1 File. Our analysis revealed the following statistically significant findings: The CRF01_AE exhibited statistically significant differences in distribution among different age groups (*P*=0.022). Significant differences in drug resistance profiles were observed among populations with distinct routes of infection (*P*=0.040). The distribution of HIV-1/AIDS patients with different routes of infection varied significantly across age groups (*P*<0.001).

**Table 3:** Distribution of two major prevalent subtypes among HIV-1/AIDS patients across different age groups.

Age	Total (%)	CRF01_AE		CRF07_BC	
		Yes	No	Yes	No
~30	20 (100.00)	4 (20.00)	16 (80.00)	14 (70.00)	6 (30.00)
31~	41(100.00)	3 (7.32)	38 (92.68)	31 (75.61)	10 (24.39)
51~	34 (100.00)	11 (32.35)	23 (67.65)	19 (55.88)	15 (44.12)
**χ^²^**		7.604		3.371	
***P* value**		0.022		0.185	

**Table 4:** Drug resistance profiles of HIV-1/AIDS patients with different routes of infection.

Route of infection	Total (%)	Drug resistance	
		Yes	No
Homosexual transmission	36 (100.00)	4 (11.11)	32 (88.89)
Heterosexual transmission	57 (100.00)	8 (14.04)	49 (85.96)
Injecting drug use	1 (100.00)	1 (100.00)	0 (0.00)
**χ^²^**		6.456	
***P* value**		0.040	

**Table 5:** Distribution of routes of infection among HIV-1/AIDS patients across different age groups.

Age	Total (%)	Homosexual transmission		Heterosexual transmission	
		Yes	No	Yes	No
~30	20 (100.00)	14 (70.00)	6 (30.00)	6 (30.00)	14 (70.00)
31~	41 (100.00)	16 (39.02)	25 (60.98)	24 (58.54)	17 (41.46)
51~	34 (100.00)	6 (17.65)	28 (82.35)	27 (81.82)	6 (18.18)
**χ^²^**		14.704		14.144	
***P* value**		<0.001		<0.001	

Further analysis was conducted using univariate and multivariate logistic regression to assess whether the statistically significant factors were potential risk factors (Tables 6 and 7). Compared with patients with HIV-1/AIDS in the -30 age group, patients with HIV-1/AIDS in the 51- age group had a 0.09 times greater risk of being infected by homosexual transmission than by non-homosexual transmission (AOR=0.09, 95%CI:0.02–0.58) and a 10.01 times greater risk of being infected by heterosexual transmission than by non-heterosexual transmission (AOR=10.01, 95%CI:1.52–35.07). 95%CI:1.52–35.07).

**Table 6:** Risk factors for Homosexual transmission in HIV-1/AIDS patients.

Variable	Crude OR (95%CI)	*P* value^a^	Adjusted OR (95%CI)	*P* value^a^
**Age**				
~30	Reference		Reference	
31~	0.27 (0.09-0.86)	0.027	0.24 (0.05-1.08)	0.063
51~	0.09 (0.03-0.34)	<0.001	0.09 (0.02-0.58)	0.012

Adjusted for Gender; Residence; Marital status; Degree of education; Genotype; Drug resistance

^a^*P* values were obtained from logistic regression.

**Table 7:** Risk factors for Heterosexual transmission in HIV-1/AIDS patients.

Variable	Crude OR (95%CI)	*P* value^a^	Adjusted OR (95%CI)	*P* value^a^
**Age**				
~30	Reference		Reference	
31~	3.29 (1.05-10.30)	0.040	3.29 (0.74-14.65)	0.117
51~	10.50 (2.85-38.63)	<0.001	10.01 (1.52-35.07)	0.017

Adjusted for Gender; Residence; Marital status; Degree of education; Genotype; Drug resistance

^a^*P* values were obtained from logistic regression.

### The distribution of DRMs

**Fig 2:**
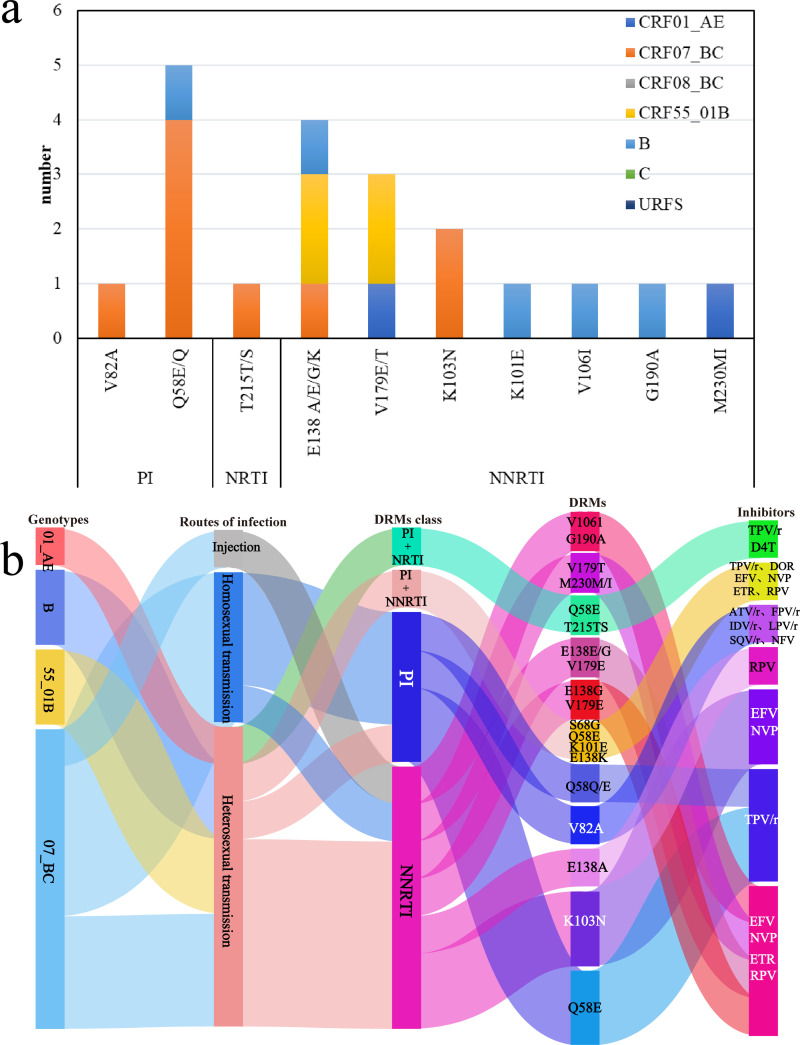
a. The distribution of DRMs among various genetic subtypes. (Note: The base map is from the China National Bureau of Surveying, Mapping and Geoinformation which provides a standard map download service, Review No. GS(2024)0650). b. The correlation between DRMs, genotypes, and the means of transmission.

DRMs were detected in 13.68% (13/95) of the successfully amplified sequences, which were distributed in CRF07_BC, CRF01_AE, CRF55_01B, and subtype B. Among them, 8 sequences were set in CRF07_BC. In detail, 7.37% (7/95) harbored NNRTI resistance-associated mutation, and 4.21% (4/95) harbored PI resistance-associated mutation. Additionally, there was one case of dual resistance to PI and NRTI, and another case of dual resistance to PI and NNRTI, respectively. In the analysis of DRMs, NNRTI exhibited the highest diversity of resistance mutations, with E138 A/E/G/K and V179E/T mutations standing out as particularly prominent. These mutations occur most frequently in the CRF55_01B genotype. In contrast, among NRTI, only one case of T215T/S mutation was detected, and this mutation was specifically present in the CRF07_BC genotype. In terms of PI, the Q58E/Q mutation dominated, with a total of five occurrences, accounting for 5.26% (5/95) of the total samples. Notably, these Q58E/Q mutations were concentrated in the CRF07_BC genotype, representing 4.21% (4/95) of the total mutation frequency (Fig 2a). Further analysis found, among the PI, intermediate resistance was exhibited by indinavir (IDV/r), lopinavir (LPV/r), and nelfinavir (NFV/r). Conversely, atazanavir (ATV/r), fosamprenavir (FPV/r), saquinavir (SQV/r), and tipranavir (TPV/r) displayed low-level resistance. In the analysis of NRTI, a solitary case of low-level resistance to stavudine (D4T) was identified. The resistance pattern of NNRTI was more intricate. Doravirine (DOR), efavirenz (EFV), etravirine (ETR), nevirapine (NVP), and rilpivirine (RPV) all exhibited low-level resistance. Notably, EFV, NVP, and RPV displayed resistance across all levels, indicating the broad and complex resistance spectrum of these NNRTI. The detailed results of this drug resistance analysis are presented (Fig 2b).

### Molecular transmission network

**Fig 3:**
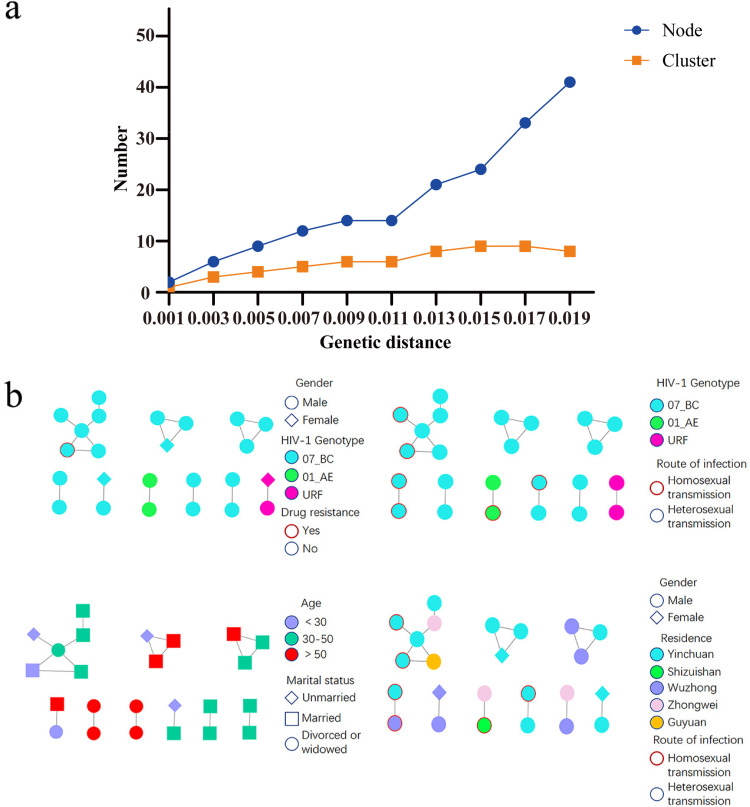
a. The number of nodes and clusters at different genetic distances. b. The molecular transmission network of newly diagnosed HIV-1 infected people before ART.

24 (25.26%, 24/95) sequences were included in 9 clusters with a genetic distance threshold of 1.5%, which identified the maximum number of clusters (Fig 3a). Among these clusters, the largest one comprises 6 nodes, and the number of nodes ranges from 1 to 4. The CRF07_BC, CRF01_AE, and URF were incorporated into the analysis of the transmission network, with male sequences comprising the majority. Heterosexual transmission was identified as the primary mode of dissemination. Notably, within the largest transmission cluster of the CRF07_BC genotype, a sequence harboring DRMs was detected. The network sequences encompass a diverse marital status distribution, with married individuals constituting the majority. The 24 sequences integrated into the molecular network are geographically distributed across five cities in NHAR, where Yinchuan accounts for the lion’s share. Additionally, there are indications of cross-city transmission patterns, (Fig 1a and Fig 3b).

### Spatial analysis

**Fig 4:**
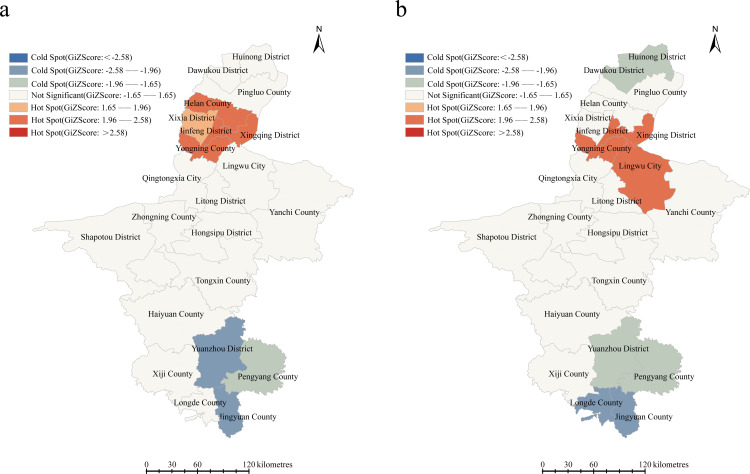
a. Hot spots of newly diagnosed HIV-1 infected people before ART at county-level in years in NHAR. (Note: The base map is from the China National Bureau of Surveying, Mapping and Geoinformation which provides a standard map download service, Review No. GS(2024)0650). b. Hot spots of newly diagnosed HIV-1 infected people with PDR at the county level in years in NHAR.

General spatial autocorrelation analysis was applied for newly diagnosed HIV-1 infected people before ART between 2020–2021. The Moran’s I value obtained was 0.31(*P* =0.0096), which indicated a significant clustering of HIV/AIDS cases among newly diagnosed adults before ART (Fig 4a). Upon analyzing the population with PDR using the same approach, we discovered that this group also displays a tendency for clustering (Moran’s I value= 0.31, *P* =0.0120). We applied local spatial autocorrelation to detect county-level hot/cold spots specifically related to the aforementioned two populations during the study period (Fig 4b). The analysis revealed that hotspots for both populations were concentrated in Yinchuan City, whereas coldspots were uniformly distributed in Guyuan City. Notably, Jinfeng District, Xingqing District, and Yongning County emerged as common hotspots, with Jingyuan County being a shared coldspot. Distinctively, Helan County and Yuanzhou District served as hotspots and coldspots, respectively, for newly diagnosed pre-treatment HIV/AIDS patients. Conversely, Lingwu City and Longde County uniquely represented hotspots and coldspots for the drug-resistant population.

## Discussion

Based on demographic parameters, we analyzed 95 newly diagnosed HIV-1 infected people before ART to reveal the prevalence and distribution characteristics of HIV-1 PDR in NHAR. This study found that 7 genotypes in newly diagnosed HIV-1 infected people before ART were epidemic in NHAR and the main subtypes are CRF07_BC and CRF01_AE, which were consistent with Yinchuan City [14] and Shaanxi Province [15], but different from Kunming City [16]and Sichuan Province [17]. The fourth molecular epidemiological survey showed that the main HIV-1 recombinant strains (such as CRF01_AE, and CRF07_BC) prevalent in China have now spread across all 31 provinces, municipalities, and autonomous regions, and their transmission has broadened significantly from IDUs to sexually transmitted populations. This study found that the young population is mainly at risk of transmission through homosexual transmission, while the elderly are at high risk of transmission through heterosexual transmission. This difference significantly reflects the profound disparities among different age groups in terms of sexual behavior patterns, risk perception abilities, and the social and cultural environment. The youth group tends to have more frequent changes of sexual partners and is inclined to expand their social circles through social media or specific social occasions such as bars, and online social platforms [18] This behavior pattern correspondingly increases their risk of exposure to HIV-1. It is worth noting that some young people have insufficient knowledge about pre-exposure prophylaxis (PrEP) and condom use, or they may have an “optimistic bias”, greatly underestimating their risk of infection, and thus failing to take adequate protective measures. In contrast, the elderly group generally lags in updating their knowledge related to HIV. Some elderly people hold the traditional concept that “there is no need to pay attention to sexual health in old age” [19], which may lead them to engage in unprotected heterosexual intercourse. Among the elderly male group, commercial sexual behavior or having multiple sexual partners may become an important route of HIV-1 infection [20]. Therefore, we need to formulate targeted prevention and control strategies according to the characteristics of the youth and the elderly populations respectively. For the youth population, we should make full use of high-density gathering areas such as schools to conduct courses on safe sexual behavior, emphasize the importance of safety protection measures such as PrEP and condoms [21], provide convenient and confidential HIV-1 self-testing tools through mobile medical platforms, reduce the barriers to testing caused by the stigmatization of men who have sex with men (MSM), and can combine short video platforms to disseminate AIDS prevention knowledge to expand the coverage [22]. For the elderly, we should make good use of senior activity centers to carry out community publicity of HIV-1, emphasize the high risk of commercial sexual behavior, and conduct regular testing.

The CRF07_BC genotype was predominantly transmitted by heterosexual and homosexual transmission, meanwhile, the transmission of new recombinant strains (such as the CRF55_01B genotype) were found. The study was consistent with the result of the fourth molecular epidemiological survey, we found that the CRF01_AE genotype was mostly transmitted by heterosexual transmission, and the CRF07_BC genotype also was transmitted by heterosexual transmission firstly, followed by homosexual transmission, only one case was Injection drug transmission. Furthermore, URF genotypes were found in this study, which revealed that the genetic subtype diversity of the HIV-1-infected patients before ART in NHAR further increased during the 2020–2021 period. This suggested a growing complexity in the HIV-1 epidemic in the region, potentially posing new challenges for prevention, diagnosis, and treatment efforts. The genetic subtype diversity in Yinchuan and Wuzhong was higher than other three cities, and the distribution of genotypes in the two cities was similar, which may be related to the people’s communication frequently between the two cities. Moreover, Yinchuan and Wuzhong have become key hubs for population migration and influx within the NHAR, attracting a large number of people from other parts of the region, which leads to the two cities incorporating HIV-1 genotypes that have emerged elsewhere.

The prevalence of PDR was 13.68% in NHAR, which reached the moderate HIV-1 resistance warning line specified by WHO (5%~15%) [23], therefore, it is necessary to further strengthen the monitoring of HIV-1 in NHAR. The result of the distribution of genotypes showed that CRF07_BC, CRF01_AE, CRF55_01B, and subtype B all generated DRMs, among of them CRF07_BC was observed the highest incidence of DRMs. The probable reasons may related to that among 95 HIV-1 infected individuals, the proportion of the CRF07_BC genotype was significantly higher than the CRF01_AE genotype, and the number of CRF07_BC genotype transmission networks and range is wide. The results found that the mutations of drug resistance genes were mainly NNRTI, higher than NRTI and PI, and the order of DRMs was consistent with that of some western provinces in China [24,25]. The incidence of DRMs in NNRTI was the highest, but the incidence of NRTI resistance mutations was low, which may be related to the use of first-line ART drugs in HIV/AIDS patients receiving ART in Ningxia, mostly two NRTI drugs combined with one NNRTI drug [26], but NNRTI had a long half-life and unit point mutation can cause HIV-1 TDR thus leading to the spread of drug-resistant strains [27]. The frequency of DRMs in the NNRTI was highest at the E138A/E/G/K. NVP and EFV were the most highly resistant and had the highest incidence of resistance. E138A/E/G/K was a weakly selected polymorphic accessory mutation, which can reduce ETR and RPV sensitivity by 2 fold [28]. The mutation frequency of Q58E/Q was higher than other mutations, both in the overall mutations and in the mutations of the PI, and it has been reported in several studies [29–31] that among the PI, the Q58E mutation was predominant and was mostly found in the CRF07_BC, which was consistent with the results of our study. This mutation was resistant to NFV and TPV and can be used as a marker for transmission of CRF07_BC. One case each of intermediate resistance to IDV, LPV, and NFV, one case each of low-level resistance to ATV, FPV, and SQV, and five cases of low-level resistance to TPV were found in the PI, suggesting that drug-resistant transmission can also occur in patients using second-line antiretroviral regimens and needs to be taken seriously for prevention and control. In this study, one case of dual resistance to NNRTI and PI and one case of dual resistance to NRTI and PI were identified, presumably reflecting the complexity of HIV-1 transmission due to frequent recombination of HIV-1 during the epidemic.

A total of 24 sequences entered the molecular network in this study, forming seven transmission clusters for the CRF07_BC genotype, one for the CRF01_AE genotype, and one for the URF genotype. The largest transmission cluster consisted of six CRF07_BC sequences, suggesting that the CRF07_BC genotype exists in more possible transmission relationships and also had a higher risk of transmission than the other genotypes. The fact that the CRF07_BC genotype had a much higher entry rate than other genotypes may be related to the high number of HIV-1 infected individuals of this genotype in the region. This also suggested that this genotype in the region had undergone more genetic mutations during transmission under the influence of multiple pressures such as environmental, drug, and individual immunity and that genetic diversity had been enriched, resulting in multiple transmission clusters. The transmission cluster of the URF genotype was formed by two cases in this study, and the two cases were a double positive couple, and the other one was MSM who was not reported in the network. Combined with the epidemiological data, it was found that both the couple had a history of non-marital commercial heterosexual contact, and the MSM had high-risk sexual intercourse with 4 other people. It was speculated that the URF genotype may have been transmitted in the high-risk population in NHAR. Therefore, the transmission and resistance of URF genotypes need to be a concern. The molecular network reveals a coexistence of sexually transmitted infections, transmitted both homosexually and heterosexually within a transmission cluster, and a direct linkage between male HIV-1 infected individuals via heterosexual transmission. This phenomenon leads us to propose the following three speculative reasons. Firstly, the research is limited to pre-treatment drug-resistant HIV-1 infected individuals, and owing to the inherent constraints of the molecular transmission network, it cannot trace the entire intermediate transmission pathways between interconnected nodes. Thus, the study may not comprehensively capture the actual transmission patterns of HIV/AIDS patients in NHAR. Secondly, social pressure and stigmatization have led to MSM feeling ashamed of their sexual identity in epidemic investigations, fearing potential discrimination if their true sexual orientation is revealed. This results in some MSM or bisexuals concealing or misreporting their sexual orientation and transmission routes. Specifically, some MSM choose to marry women to conceal their true identity, putting them in a high-risk category for HIV-1 transmission. Therefore, it is crucial to strengthen HIV prevention and control efforts targeting this population, aiming to promptly identify and interrupt their transmission pathways. Lastly, the lack of public knowledge regarding HIV transmission routes and prevention awareness also contributes to this phenomenon. Without relevant knowledge, individuals often fail to accurately determine their transmission routes and sources. Consequently, enhancing public awareness of HIV prevention, coupled with the promotion and education of relevant knowledge, is of utmost importance in containing the spread of HIV-1. The study found that the largest number of married HIV-1 infected individuals entered the network, and the largest number were between the ages of 30 and 50. It is hypothesized that this is because married people make up the highest proportion of the total population included in the study and are at a higher frequency and risk of having high-risk sexual interactions when they are sexually active. On this basis, if the rate of notification to sexual partners is low and partners are unaware of their infection, it is more likely to lead to HIV-1 transmission. Therefore, efforts should be made to promote HIV prevention, reduce people’s resistance to HIV, promote the rate of HIV notification, and also focus on the use of prophylaxis such as condoms, pre-exposure prophylaxis, and post-exposure prophylaxis. Both genotypes CRF07_BC and CRF01_AE show clusters of divorced/widowed middle-aged and older people aged >50 years. This situation may be related to the lack of emotional care for widows and orphans and their susceptibility to high-risk sexual interactions, suggesting that it is not uncommon for middle-aged and older adults to engage in high-risk intercourse for HIV transmission due to their emotional status, resulting in the transmission of HIV-1 strains.

Spatial autocorrelation analysis revealed that cold spots for both newly diagnosed HIV-1 infected people before ART and those who have developed drug resistance are concentrated in the southern mountainous regions of NHAR. This distribution may be attributed to the inconvenience of transportation in these areas, which hinders the spread of HIV-1. Conversely, Yinchuan City emerges as a hotspot for pre-treatment HIV-1 infections, likely due to its status as the capital city of NHAR, where advanced medical resources facilitate the timely identification of HIV/AIDS patients. Notably, The districts and counties under the jurisdiction of Yinchuan City are also hotspots for transmitted drug resistance. This can be explained by the increasing demand for labor in major Chinese cities, prompting Yinchuan, as an economically developed city with convenient transportation in NHAR, to become a preferred destination for migrant workers. Its relatively robust economic growth may have contributed to an increase in the number of sexual service establishments, both of which are significant factors in amplifying the risk of drug resistance transmission. This phenomenon is further corroborated by molecular transmission networks, wherein the dominant cluster of resistant strains traces back to Yinchuan. Furthermore, the inter-city transmission of viral strains may lead to the emergence of novel recombinant subtypes through recombination with locally prevalent subtypes, thereby enhancing the diversity of HIV quasispecies in these regions. As a result, public health professionals and policymakers can leverage the aforementioned evidence to devise tailored strategies for various regions, prioritizing the allocation of public health resources towards these hotspots. This will enhance resource utilization efficiency and effectively curb the spread of HIV-1 drug resistance.

This study also has certain limitations. According to the China Health and Family Planning Statistics Yearbook and reports from the Ningxia Center for Disease Control and Prevention, Ningxia is a low HIV prevalence region, with a local HIV incidence rate of 3.8 per 100,000. During the study period (2020–2021), China’s national “Test and Treat” policy drastically reduced the number of newly reported HIV-1/AIDS patients before ART. As a result, the number of newly reported HIV-1/AIDS patients before ART was minimal, which is the main reason for the relatively small sample size in this study. This constraint may introduce bias and elevate the risk of Type II errors, where true associations might be overlooked due to insufficient statistical power. Furthermore, the sample may not fully represent the broader patient population, potentially leading to an overestimation of drug resistance prevalence and incomplete characterization of molecular transmission networks. These limitations necessitate a cautious interpretation of our findings and underscore the importance of future large-scale, comprehensive studies to validate and extend our observations. Nevertheless, given China’s upcoming adjustments to ART policies starting in 2025, this study provides critical preliminary insights to guide subsequent ART policy revisions in Ningxia. In future research, we will prioritize the collection of samples and data from newly reported pre-treatment HIV-1/AIDS patients to enhance the generalizability and public health relevance of our conclusions.

## Conclusions

In summary, this study revealed a further increase in HIV-1 diversity within NHAR, with the overall prevalence of PDR at a moderate level. The phenomena of PDR transmission and cross-regional dissemination were observed. From a spatial perspective, the distribution of cases exhibits a distinct geographical trend, primarily concentrated in districts and counties under the jurisdiction of Yinchuan City. Therefore, to mitigate the emergence and spread of drug-resistant strains, it is imperative to strengthen PDR testing and adjust ART protocols accordingly. By integrating epidemiological investigations with molecular surveillance techniques, we can devise targeted strategies based on spatial distribution characteristics, thereby reducing the occurrence and dissemination of resistant virus variants.

## Supporting information

S1 File This is a set of supplementary tables. (DOCX)

## Abbreviations

NHAR: Ningxia Hui Autonomous Region
PDR: pre-treatment drug resistance
ART: antiretroviral therapy
DRMs: Drug resistance mutations
NFATP: National Free Antiretroviral Treatment Program
VL: viral load
TDR: transmitted drug resistance
PLWH: people living with HIV-1
URFs: unique recombination forms
IDV/r: indinavir
LPV/r: lopinavir
NFV/r: nelfinavir
ATV/r: atazanavir
HIV-1: human immunodeficiency virus type 1
FPV/r: fosamprenavir
SQV/r: saquinavir
TPV/r: tipranavir
D4T: stavudine
DOR: Doravirine
EFV: efavirenz
ETR: etravirine
NVP: nevirapine
RPV: rilpivirine
IDUs: injection drug users
