# Supplementary material for: Epidemiological and spatial analysis of newly diagnosed HIV-1/AIDS patients before antiretroviral therapy in Ningxia from 2020 to 2021
Source: PLoS One. 2025 Apr 22;20(4):e0322389. doi: 10.1371/journal.pone.0322389 (PMC12013917; doi:10.1371/journal.pone.0322389)
Supplement: S1 File — (DOCX) [file pone.0322389.s001.docx]

Supplementary tables

| S1 Table Distribution of two major prevalent subtypes among HIV-1/AIDS patients with different routes of infection | | | | | |
| --- | --- | --- | --- | --- | --- |
| **Route of infection** | **Total(%)** | **CRF01_AE** | | **CRF07_BC** | |
|  |  | Yes | No | Yes | No |
| Homosexual transmission | 36(100.00) | 6 (16.67) | 30 (39.47) | 27 (75.00) | 9 (25.00) |
| Heterosexual transmission | 57(100.00) | 12 (21.05) | 45 (78.95) | 35 (61.40) | 22 (38.60) |
| Injecting drug use | 1 (100.00) | 0 (0.00) | 1 (100.00) | 1 (100.00) | 0 (0.00) |
| χ² |  | 0.514 | | 2.323 | |
| *P* value |  | 0.774 | | 0.310 | |

| S2 Table Drug resistance profiles of the two major prevalent HIV-1 subtypes | | | | | |
| --- | --- | --- | --- | --- | --- |
| **Route of infection** | **Total(%)** | **CRF01_AE** | | **CRF07_BC** | |
|  |  | Yes | No | Yes | No |
| Yes | 13(100.00) | 1 (7.69%) | 12 (92.31) | 8 (61.54) | 5 (38.46) |
| No | 82(100.00) | 17 (20.73) | 65 (79.27) | 56 (68.29) | 26 (31.71) |
| χ² |  | 1.242 | | 0.233 | |
| *P* value |  | 0.265 | | 0.629 | |

| S3 Table Drug resistance profiles of HIV-1/AIDS patients across different age groups | | | |
| --- | --- | --- | --- |
| **Age** | **Total (%)** | **Drug resistance** | |
|  |  | Yes | No |
| ~30 | 20 (100.00) | 3 (15.00) | 17 (85.00) |
| 31~ | 41 (100.00) | 4 (9.76) | 37 (90.24) |
| 51~ | 34 (100.00) | 6 (17.65) | 28 (82.35) |
| χ² |  | 1.017 | |
| *P* value |  | 0.601 | |
